# MvaT binds to the P*_exsC_* promoter to repress the type III secretion system in *Pseudomonas aeruginosa*

**DOI:** 10.3389/fcimb.2023.1267748

**Published:** 2023-11-06

**Authors:** Liwen Yin, Qi Liu, Xiaolei Pan, Chenjing Lv, Yuxi Bai, Fang Bai, Zhihui Cheng, Weihui Wu, Un-Hwan Ha, Yongxin Jin

**Affiliations:** ^1^ State Key Laboratory of Medicinal Chemical Biology, Key Laboratory of Molecular Microbiology and Technology of the Ministry of Education, Department of Microbiology, College of Life Sciences, Nankai University, Tianjin, China; ^2^ Department of Biotechnology and Bioinformatics, Korea University, Sejong, Republic of Korea

**Keywords:** *P. aeruginosa*, T3SS, MvaT, transcriptional regulation, P*_exsC_*, P*_exsA_*

## Abstract

*Pseudomonas aeruginosa* is an opportunistic human pathogen capable of causing a variety of acute and chronic infections. Its type III secretion system (T3SS) plays a critical role in pathogenesis during acute infection. ExsA is a master regulator that activates the expression of all T3SS genes. Transcription of *exsA* is driven by two distinct promoters, its own promoter P*_exsA_* and its operon promoter P*_exsC_*. Here, in combination with a DNA pull-down assay and mass spectrometric analysis, we found that a histone-like nucleoid-structuring (H-NS) family protein MvaT can bind to the P*_exsC_* promoter. Using EMSA and reporter assays, we further found that MvaT directly binds to the P*_exsC_* promoter to repress the expression of T3SS genes. The repression of MvaT on P*_exsC_* is independent of ExsA, with MvaT binding to the -429 to -380 bp region relative to the transcription start site of the *exsC* gene. The presented work further reveals the complex regulatory network of the T3SS in *P. aeruginosa*.

## Introduction

*Pseudomonas aeruginosa* is a Gram-negative opportunistic pathogen that causes a variety of infections in immunocompromised individuals (Reynolds and Kollef, 2021). To successfully establish infections in the host, *P. aeruginosa* expresses a series of virulence determinants, including the type III secretion system (T3SS). The T3SS is a needle-like machinery deployed by *P. aeruginosa* to inject toxic proteins directly into host cells, including four well-known effectors ExoS, ExoT, ExoY, and ExoU (Jouault et al., 2022). It plays a critical role in the colonization and pathogenesis of *P. aeruginosa* during human and animal infections (Holder et al., 2001).

The regulation of the T3SS is complicated and involves varieties of factors in *P. aeruginosa* (Williams McMackin et al., 2019a). ExsA is a master regulator that promotes expression of the whole T3SS regulon by binding to the promoters of T3SS. ExsA also activates its own expression via the P*_exsC_* promoter which drives an operon consisting of *exsC*, *exsE*, *exsB* and *exsA* genes (Williams McMackin et al., 2019a). In addition to the operon promoter (P*_exsC_*), the expression of *exsA* is also stimulated by its own promoter P*_exsA_*, which is located at the intergenic region between *exsB* and *exsA* (Liang et al., 2014). Many regulatory players control the expression of the T3SS by directly or indirectly regulating *exsA* transcription and/or translation. Although P*_exsA_* displays a relatively weak promoter activity compared to the P*_exsC_*, several regulatory factors have been found to modulate P*_exsA_* promoter activity. Vfr, the global regulator of virulence gene expression, activates T3SS gene expression via direct binding and activation of the P*_exsA_* promoter (Marsden et al., 2016). MvaT and MvaU, histone-like nucleoid-structuring (H-NS) DNA-binding proteins, repress T3SS gene expression by direct binding and silencing of the P*_exsA_* promoter (Williams McMackin et al., 2019b). VqsM, the AraC-family transcription factor, directly binds to the promoter region of P*_exsA_* to stimulate T3SS (Liang et al., 2014). Fis, a versatile DNA binding protein, specifically binds to the intergenic region between *exsB* and *exsA* to act in transcription elongation from *exsB* to *exsA* (Deng et al., 2017). However, except for PsrA, the factors contributing to T3SS expression by controlling P*_exsC_* promoter activity remain elusive.

In this study, combining a DNA pull-down assay and mass spectrometric analysis, we identified candidate regulators that bind to and regulate the P*_exsC_* promoter. We demonstrated that MvaT also directly binds to the P*_exsC_* promoter to repress the expression of T3SS genes. The repressive function of the MvaT on P*_exsC_* is independent of ExsA, with MvaT binding to the -429 to -380 bp region relative to the transcription start site of the *exsC* gene. The presented work further reveals the complex regulatory network of the T3SS in *P. aeruginosa*.

## Materials and methods

### Bacterial strains, plasmids and primers

The bacterial strains, plasmids and primers used in this study are listed in **Supplementary Tables 1**, **2**. Bacterial cells were grown in Luria–Bertani (LB) medium (5 g/L NaCl, 5 g/L yeast extract, and 10 g/L tryptone) or on LB agar plates (LB medium containing 15 g/L agar) at 37 °C. To maintain plasmids, appropriate antibiotics were supplemented into the medium at the following concentrations: for *P. aeruginosa*, tetracycline at 50 μg/mL and carbenicillin at 150 μg/mL; for *E. coli*, tetracycline at 10 μg/mL, kanamycin at 25 µg/mL, and ampicillin at 100 μg/mL. When needed, IPTG (isopropyl β-D-1-thiogalactopyranoside) was added to the medium at a final concentration of 1 mM. For inducing T3SS of *P. aeruginosa*, EGTA was added to the medium at a final concentration of 5 mM.

### Construction of plasmids and bacterial strains

For complementation of the *mvaT* gene, the *mvaT* gene was amplified by PCR using specific primers (**Supplementary Table 2**) with PAK genomic DNA as the template. The resulting PCR product was digested with *Bam*HI-*Hin*dIII and then cloned into pMMB, generating pMMB-*mvaT*. P*_exsC_* mut-*lacZ* (*Eco*RI-*Bam*HI), pMMB-*exsA*-His (*Eco*RI-*Hin*dIII) and pET28a-*mvaT* (*Nco*I-*Xho*I) were constructed with similar strategies. To delete the *mvaT* gene, two DNA fragments upstream and downstream of the *mvaT* gene were amplified by PCR using the specific primer pairs *mvaT*-UF/UR and *mvaT*-DF/DR (**Supplementary Table 2**), digested with appropriate restriction enzymes and directionally cloned into pEX18Tc, resulting in the deletion construct pEX18Tc-*mvaT*. To obtain the *mvaT* deletion mutant in the PAK and PAKΔ*exsA* strains, pEX18Tc-*mvaT* was transferred into the corresponding bacterial cell via conjugation, and gene deletion was performed with a SacB-based strategy as previously described (Hoang et al., 1998).

### DNA pull-down assay

The DNA pull-down assay was carried out as described previously with minor modifications (Dolan et al., 2020; Modrzejewska et al., 2021). A biotinylated DNA fragment containing the P*_exsC_* promoter was amplified by PCR using primers P*_exsC_*-biot-F/R (**Supplementary Table 2**) with PAK genomic DNA as a template. The amplified DNA segment was purified and incubated with 100 μL of streptavidin magnetic beads (Streptavidin Mag Sepharose, Cytiva) at room temperature for 20 min. Then, the magnetic beads were washed twice with TBS buffer (50 mM Tris, 150 mM NaCl, pH 7.5) to remove the unbound DNA. In parallel, PAK cells from 200 mL cultures at the exponential phase were harvested by centrifugation at 8,000 × g for 10 min, resuspended in 3 mL TBS buffer and lysed by sonication on ice. After centrifugation at 12,000 × g for 10 min at 4°C, the supernatant was mixed with the immobilized DNA fragments on the magnetic beads and incubated at 4°C for 1 h. After that, the beads were washed three times with TBS buffer to remove nonspecifically bound proteins, and the DNA-bound proteins were released by treatment with increasing concentrations of NaCl buffer (0.2 M, 0.5 M, 1 M). PAKΔ*exsA*/pMMB-*exsA*-His lysates incubated with P*_exsC_*-coupled magnetic beads served as a positive control, while PAK cell lysates incubated with DNA-uncoupled magnetic beads served as a negative control. The eluates from the ExsA-His-overproducing strain were subjected to Western blot analysis using anti-His antibodies to confirm the predicted binding of the ExsA-His protein to the P*_exsC_* promoter, validating the pull-down procedure. The eluates from the PAK strain with or without DNA-coupled beads were examined by SDS-PAGE and stained with Coomassie blue. The protein band of interest (the band observed in DNA-coupled beads, but not in the negative control) was excised from SDS-PAGE gel (from 0.2 M NaCl buffer elution) and identified by mass spectrometry analysis.

### β-galactosidase assay

The β-galactosidase assay was carried out as described previously with minor modifications (Miller, 1972). Overnight cultures of bacteria were 50-fold diluted into fresh LB medium with 0 (for *E. coli* or *P. aeruginosa* T3SS non-inducing condition) or 5 mM EGTA (for *P. aeruginosa* T3SS inducing condition) and grown at 37°C with continuous agitation at 200 rpm. When the OD_600_ reached 1.0, 500 μL bacterial cells were collected by centrifugation and resuspended in 1.5 mL Z-buffer (60 mM Na_2_HPO_4_, 60 mM NaH_2_PO_4_, 50 mM β-mercaptoethanol, 10 mM KCl, 1 mM MgSO_4_). One milliliter suspension was used to measure the OD_600_, and 10 μL chloroform and 10 μL 0.1% SDS were added to the remaining 500 μL suspensions, followed by vortexing for 10 s. After that, 100 μL ONPG (4 mg/mL) was added to the reaction mixture and incubated at 37°C. The reaction was stopped by the addition of 500 μL of 1 M Na_2_CO_3_. The reaction time was recorded, and OD_420_ was measured after centrifugation at 16,000 × g for 5 min. β-galactosidase activity (Miller units) was calculated as 1000×OD_420_/T_min_/0.5/OD_600_; T_min_ represents reaction time (minutes).

### Western blot assay

Overnight bacterial cultures were diluted 50-fold into 3 mL fresh LB medium containing 0 or 5 mM EGTA and then grown to an OD_600_ of 1.0 with continuous shaking at 200 rpm. Equivalent numbers of bacteria were harvested by centrifugation at 12,000 × g for 3 min. Samples from bacterial cells and supernatant were mixed with loading buffer, boiled for 10 min at 99 °C, and then separated on a 12% SDS-PAGE gel. After transfer onto a polyvinylidene difluoride (PVDF) membrane, the proteins were probed with the primary antibody against ExoS or RpoA (RNAP, Abcam) for 1 h at room temperature and then with the corresponding horseradish peroxidase (HRP)-conjugated secondary antibody, anti-rabbit IgG (Promega) or anti-mouse IgG (Promega), for 1 h at room temperature. All antibodies were 2000-fold diluted into PBST buffer with 5% non-fat milk. The signals were detected using an ECL Plus kit (Millipore) and visualized in a Bio-Rad molecular imager (ChemiDoc XRS+).

### Protein purification

To express the recombinant C-terminal His-tagged MvaT, the *E. coli* strain BL21 (DE3) containing pET28a-*mvaT* was cultured at 37°C to an OD_600_ of 0.6. Overexpression of the recombinant MvaT-His protein was induced by the addition of 1 mM IPTG and further grown overnight at 16°C. Harvested cells were resuspended in lysis buffer (50 mM sodium phosphate, 0.3 M NaCl, pH 8.0) and lysed by sonication on ice. After centrifugation at 12,000 × g for 10 min, the supernatant was incubated with Ni-NTA resin (Qiagen) at 4°C for 1 h. Nonspecific binding proteins were washed away with lysis buffer containing 20 mM imidazole, and MvaT-His was eluted with the lysis buffer containing 200 mM imidazole. The purified protein was examined by SDS-PAGE and stained with Coomassie blue.

### Electrophoretic mobility shift assays (EMSA)

EMSA was carried out following a previous description with minor modifications (Williams McMackin et al., 2019b). Briefly, the P*_exsC_* DNA fragment was amplified by PCR using specific primers (**Supplementary Table 2**). Fifty or 20 nanogram DNA probe was incubated with increasing concentrations of purified MvaT-His protein in a 20-μL reaction (20 mM Tris, 100 mM KCl, 1 mM DTT, 10% glycerol, pH 7.5) on ice for 30 min. Each sample was loaded onto an 8% or 12% native polyacrylamide gel in 0.5 × TBE (Tris-borate-EDTA) buffer (44.5 mM Tris base, 44.5 mM boric acid, 1 mM EDTA, pH 8.0) and electrophoresed on ice at 10 mA for 1 h. The gel was then stained in 0.5 × TBE buffer containing 0.5 μg/mL ethidium bromide (EB) and imaged with a molecular imager ChemiDoc XRS+ (Bio-Rad, CA, USA).

## Results

### Candidate proteins bound to the P*_exsC_* promoter

**Figure 1:**
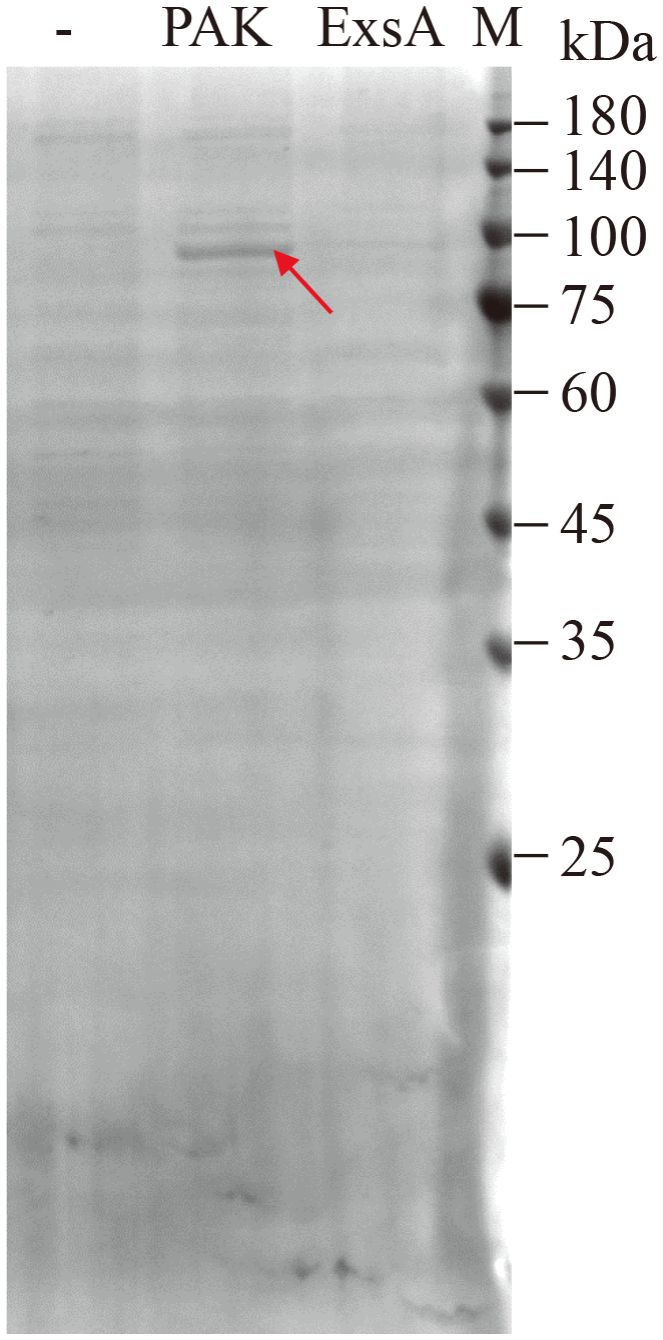
Identification of candidate proteins binding to the P*_exsC_* promoter. Biotin-labeled P*_exsC_* promoter fragment was incubated with cell lysates of PAK or PAKΔ*exsA*/pMMB-*exsA*-His, purified with streptavidin-coated beads, separated by SDS-PAGE, and stained with Coomassie blue. The protein band different from the control sample (-, cell lysates of PAK without DNA fragment) is indicated with an arrow, excised, and analyzed by LC-MS/MS.

To identify proteins that can modulate *exsA* transcription via the P*_exsC_* promoter and consequently T3SS expression, we used a biotin-labeled P*_exsC_* promoter fragment as bait in a DNA pull-down assay using *P. aeruginosa* PAK cell lysates. Proteins copurified with the P*_exsC_* fragment by streptavidin-coated beads were separated by SDS-PAGE and stained with Coomassie blue. As shown in **Figure 1**, a specific protein band was detected in the samples in the presence but not in the absence of the P*_exsC_* fragment. As a positive control, lysates of bacterial cells overproducing ExsA (PAKΔ*exsA*/pMMB-*exsA*-His) were pulled down by the P*_exsC_* fragment and subjected to Western blot against His antibody. ExsA-His was easily detected (**Supplementary Figure 1**), validating our approach. Mass spectrometric analysis of the protein band collection revealed a mixture of 65 proteins (**Supplementary Table 3**). Among them, MvaT, the histone-like nucleoid-structuring DNA-binding protein, was detected. Interestingly, a previous study showed that MvaT regulates the T3SS via direct silencing of the P*_exsA_* promoter (Williams McMackin et al., 2019b).

### MvaT binds to P*_exsC_* to repress its activity

**Figure 2:**
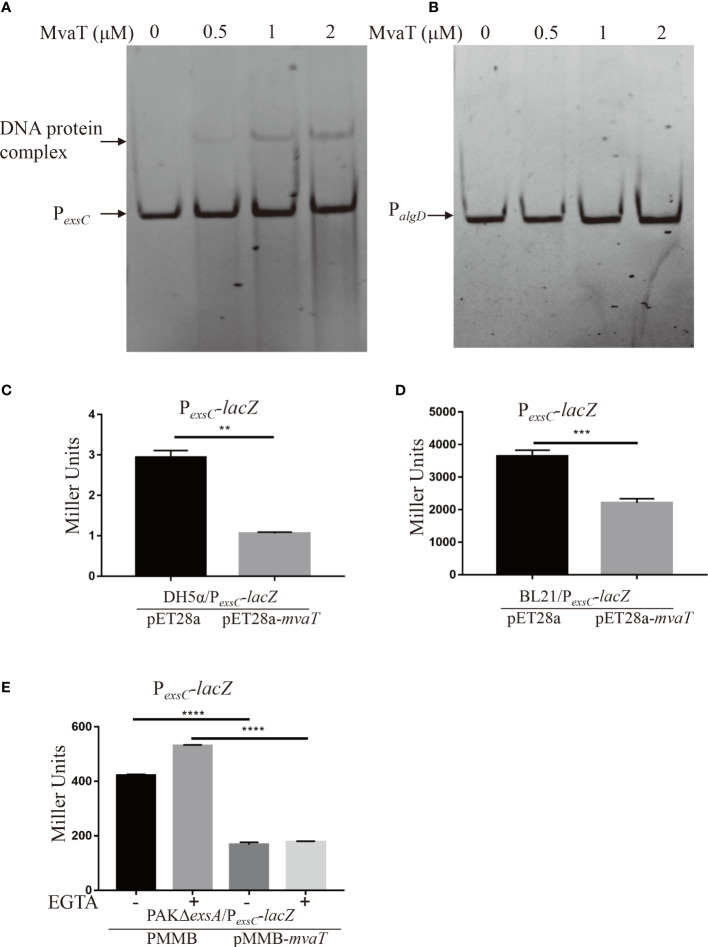
MvaT binds to and represses P*_exsC_* directly. **(A, B)** MvaT binds to the P*_exsC_* but not P*_algD_* promoter. The DNA probe (20 ng) was incubated with 0, 0.5, 1 or 2 µM MvaT on ice for 30 min. The shifted band is indicated by an arrowhead. **(C-E)** To test the repression on promoter activity of P*_exsC_* by MvaT, β-galactosidase activity assays were carried out in the indicated stain backgrounds. DH5α/pET28a and DH5α/pET28a-*mvaT* **(C)**, BL21/pET28a and BL21/pET28a-*mvaT* **(D)**, and PAKΔ*exsA*/pMMB and PAKΔ*exsA*/pMMB-*mvaT* **(E)** containing the P*_exsC_*-*lacZ* transcriptional reporter plasmids were grown to an OD_600_ of 1.0 in LB **(C, D)** or LB with (+) or without (-) 5 mM EGTA **(E)** and subjected to β-galactosidase assays. Each assay was performed in triplicate, and the error bars indicate standard deviations. **P < 0.01, ***P < 0.001, ****P < 0.0001, by Student’s *t* test.

To determine whether MvaT, identified in the pull-down analysis, can indeed bind to the P*_exsC_* promoter, we carried out electrophoretic mobility shift assays (EMSAs) using purified MvaT-His and DNA fragments corresponding to the P*_exsC_* promoter region. The DNA fragments corresponding to P*_exsC_*, but not the negative control P*_algD_*, were shifted upon incubation with MvaT-his (**Figures 2A, B**), indicating that MvaT directly binds to the promoter of P*_exsC_* in *P. aeruginosa*.

To further examine the influence of MvaT on the activity of the P*_exsC_* promoter, the *mvaT* gene was expressed under the control of P_T7_ in vector pET28a and introduced into DH5α and BL21 harboring the P*_exsC_*-*lacZ* transcriptional fusion reporter plasmid. Measurement of β-galactosidase activity showed that expression of MvaT significantly repressed P*_exsC_* activity in both DH5α and BL21 (**Figures 2C, D**). However, the β-galactosidase activity of P*_exsC_*-*lacZ* in BL21 is much higher than that in DH5α, which may be due to the absence of OmpT protease in BL21. These results suggested that MvaT acted directly as a repressor of the P*_exsC_* promoter. Furthermore, we wanted to investigate whether MvaT modulates P*_exsC_* activity in *P. aeruginosa*. A previous study demonstrated that MvaT binds to and represses the P*_exsA_* promoter (Williams McMackin et al., 2019b). To exclude the effect of MvaT on P*_exsC_* activity derived from the altered ExsA protein, P*_exsC_*-*lacZ* and pMMB-*mvaT* were co-introduced into the PAKΔ*exsA* strain. Consistent with the P*_exsC_*-*lacZ* reporter results in *E. coli*, expression of MvaT resulted in significant decreases in β-galactosidase activity under both T3SS inducing and noninducing conditions (**Figure 2E**). All these results demonstrated that MvaT represses the P*_exsC_* promoter activity directly.

### MvaT represses the T3SS through P*_exsC_*

**Figure 3:**
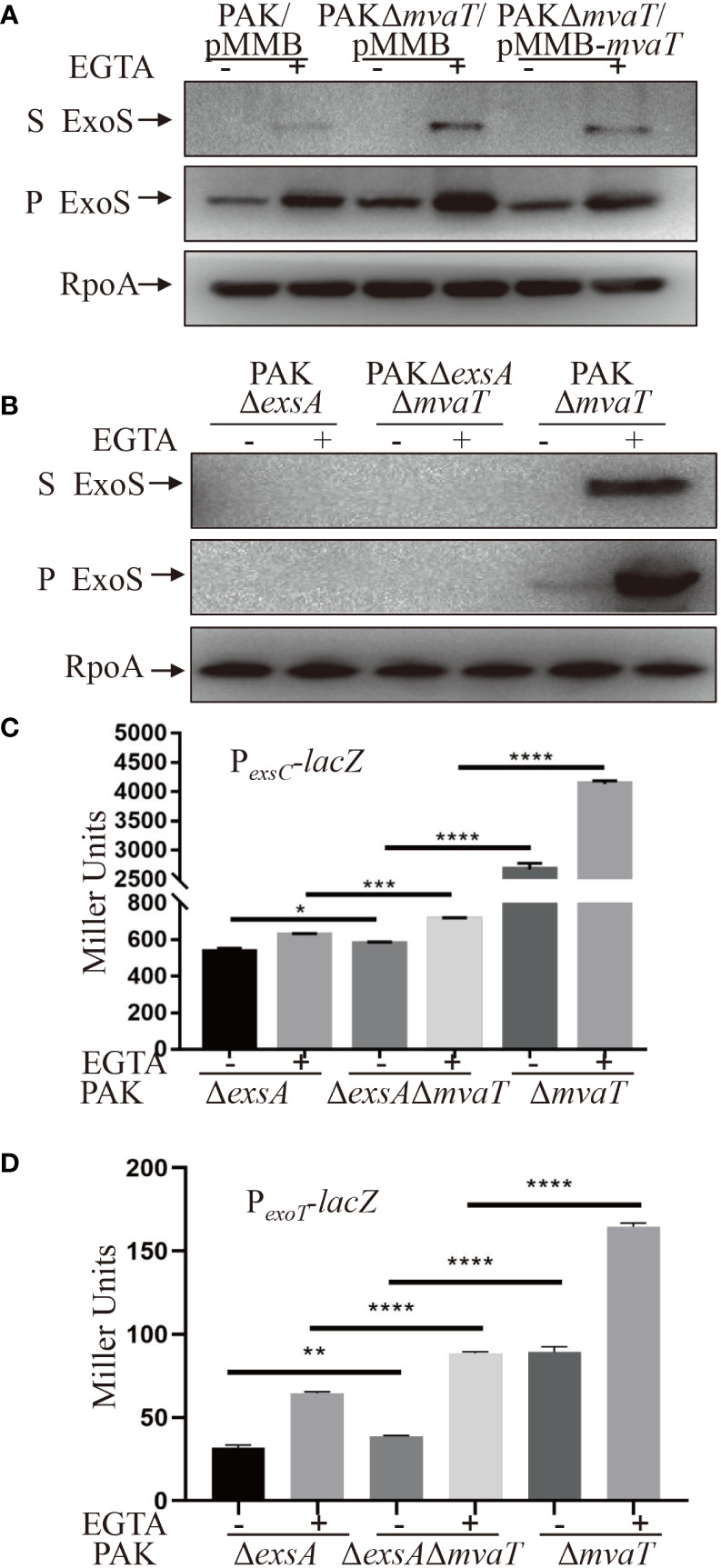
MvaT represses the T3SS independent of ExsA. **(A, B)** Expression and secretion of ExoS in the indicated strains. Bacterial cells were grown to an OD_600_ of 1.0 in LB with (+) or without (-) 5 mM EGTA. 1 mM IPTG was added to induce expression of *mvaT* in **(A).** Proteins in supernatants (S) and pellets (P) from equivalent bacterial cells were separated by 12% SDS-PAGE gels and probed with anti-ExoS antibody or anti-RpoA antibody. **(C, D)** Indicated strains containing the P*_exsC_*-*lacZ* or P*_exoT_*-*lacZ* transcriptional reporter plasmid were grown to an OD_600_ of 1.0 in LB with 0 (-) or 5 mM (+) EGTA and subjected to β-galactosidase assays. Each assay was performed in triplicate, and the error bars indicate standard deviations. *P < 0.05, **P < 0.01, ***P < 0.001, ****P < 0.0001, by Student’s *t* test.

To determine the influence of MvaT on T3SS gene expression, we generated a *mvaT* clear deletion mutant and monitored the ExoS amounts using Western blot assay. Strains were cultured under T3SS-inducing (with 5 mM EGTA) and noninducing (without EGTA) conditions for T3SS gene expression. As shown in **Figure 3**, the expression and secretion of ExoS were highly induced under inducing conditions in both the wild-type PAK and *mvaT* mutant strains. Deletion of *mvaT* resulted in increased expression and secretion of ExoS compared to the wild-type PAK strain, and complementation with a *mvaT* gene recovered the expression and secretion of ExoS in the *mvaT* mutant background (**Figure 3A**).

To examine the effect of MvaT on ExoS expression and secretion independent of ExsA, Western blot was carried out to compare the ExoS amounts between PAKΔ*exsA* and PAKΔ*exsA*Δ*mvaT*. However, in the absence of *exsA*, ExoS was undetectable in both PAK and PAKΔ*mvaT* (**Figure 3B**). Since the β-galactosidase activity of P*_exsC_*-*lacZ* was detectable in the absence of *exsA* in PAK (**Figure 2E**), we further examined and compared the P*_exsC_* promoter activities. As shown in **Figure 3C**, *mvaT* deletion resulted in increased β-galactosidase activity in PAKΔ*exsA/*P*_exsC_-lacZ* under both T3SS-inducing and noninducing conditions. In addition, the PAKΔ*mvaT* strain displayed a much higher β-galactosidase activity than the PAKΔ*exsA*Δ*mvaT* strain (**Figure 3C**), hinting at the master activator role of ExsA and a repressive role of MvaT on P*_exsA_* promoter (Williams McMackin et al., 2019b). Overall, these data suggest that MvaT represses P*_exsC_* transcription independent of ExsA. Furthermore, we examined the promoter activity of another T3SS effector ExoT with P*_exoT_*-*lacZ* transcriptional fusion reporter plasmid (Ha and Jin, 2001). Consistent with the P*_exsC_* promoter activities, absence of *mvaT* resulted in increased β-galactosidase activities of P*_exoT_*-*lacZ* in the PAKΔ*exsA* strain under both T3SS inducing and non-inducing conditions (**Figure 3D**).

### MvaT binds to -429 to -380 bp relative to the transcription start site of the *exsC* gene

**Figure 4:**
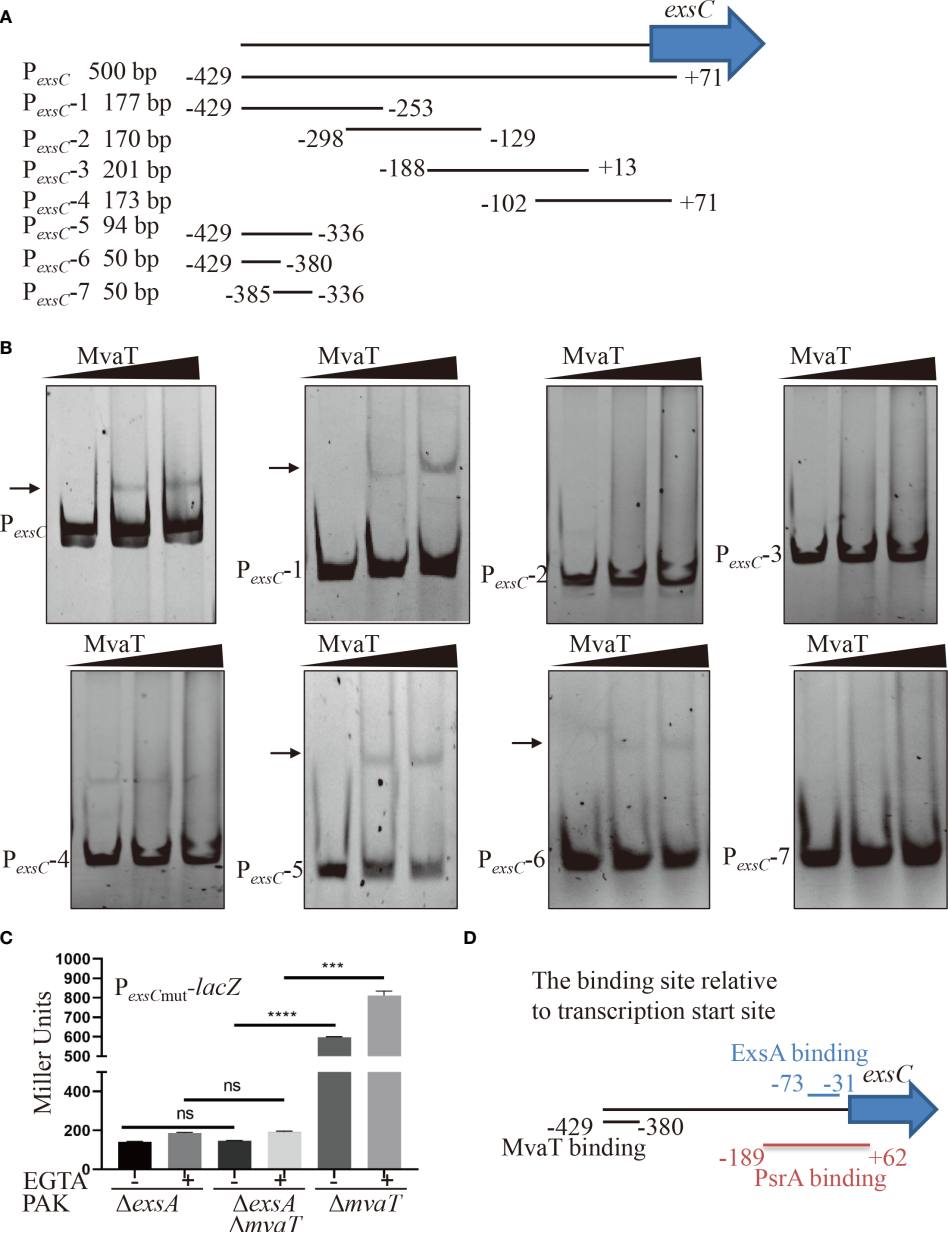
Identification of the binding region of MvaT in the promoter of P*_exsC_*. **(A)** Diagram of the DNA probe in the P*_exsC_*. The number indicated the position relative to the transcription start site of *exsC* gene. **(B)** Different DNA fragments within P*_exsC_* were incubated with 0, 2 or 4 µM MvaT on ice for 30 min. The shifted band is indicated by an arrowhead. **(C)** Indicated strains containing the P*_exsC_*_mut_-*lacZ* transcriptional reporter plasmid were grown to an OD_600_ of 1.0 in LB with 0 (-) or 5 mM (+) EGTA and subjected to β-galactosidase assays. Each assay was performed in triplicate, and the error bars indicate standard deviations. ns, not significant, ***P < 0.001, ****P < 0.0001, by Student’s *t* test. **(D)** The binding model of MvaT and previously identified ExsA and PsrA (Hovey and Frank, 1995; Shen et al., 2006).

ExsA binds to upstream of the transcriptional start site and facilitates transcription by recruiting RNAP-σ^70^ to the P*_exsC_* promoter (Vakulskas et al., 2009). MvaT represses P*_exsC_* promoter activity independent of ExsA. Therefore, we wanted to determine the binding site of MvaT on the P*_exsC_* promoter. A variety of DNA fragments within P*_exsC_* (**Figure 4A**) were PCR amplified and used as DNA probes in the EMSA. As shown in **Figure 4B**, MvaT binds to the DNA fragments containing -429 to -380 bp relative to the transcription start site of the *exsC* gene. To examine the function of the MvaT binding site in the MvaT-mediated repression of *exsC* gene, we constructed an additional P*_exsC_*_mut_-*lacZ* transcriptional fusion, where a 397-bp fragment upstream of the *exsC* coding region without the MvaT binding site was fused to the promoterless *lacZ* gene. As shown in **Figure 4C**, removal of the MvaT binding site resulted in no difference of the β-galactosidase activity between PAKΔ*exsA*Δ*mvaT* and PAKΔ*exsA* strain. This result further supports that MvaT binds to and represses P*_exsC_* independent of the master activator ExsA.

## Discussion

**Figure 5:**
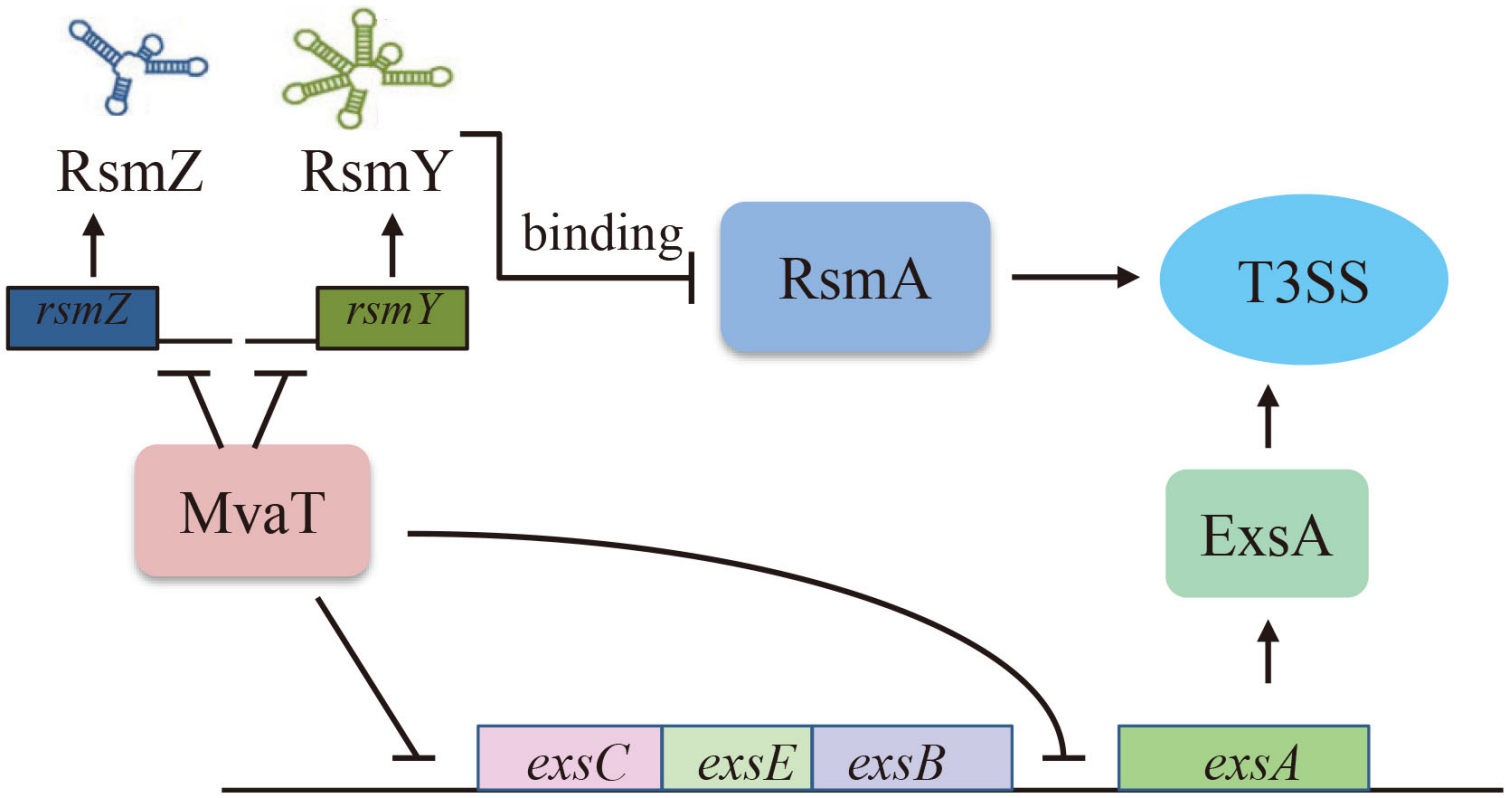
Proposed model of MvaT-mediated T3SS regulation in *P. aeruginosa*. MvaT regulates T3SS via direct binding and silencing the P*_exsC_* and P*_exsA_* promoters, as well as RsmY/Z-RsmA pathway indirectly (Williams McMackin et al., 2019b).

MvaT is a member of the H-NS family of proteins, which are encoded by many Gram-negative bacteria, including *Pseudomonas* (Tendeng et al., 2003). In *P. aeruginosa*, MvaT serves as a global regulator and regulates the expression of more than 150 genes (Vallet et al., 2004; Castang et al., 2008), including virulence factor-encoding genes (Vallet et al., 2004). Similar to other H-NS-like proteins in enteric bacteria, MvaT acts as a transcriptional silencer of foreign DNA by preferentially binding to AT-rich elements of chromosomes in *P. aeruginosa* (Castang et al., 2008). MvaT negatively controls the expression of fimbrial *cup* gene clusters and biofilm formation (Vallet et al., 2004; Vallet-Gely et al., 2005). MvaT modulates chloramphenicol resistance in *P. aeruginosa* by controlling the expression of the multidrug efflux pump MexEF-OprN (Westfall et al., 2006). Recently, MvaT was shown to control T3SS gene expression via direct silencing of the P*_exsA_* promoter and indirectly via the RsmY/Z-RsmA pathway (Williams McMackin et al., 2019b). In this study, we found that MvaT also directly binds to the P*_exsC_* promoter to repress the expression of T3SS genes. Combining these findings, a model has been proposed for the MvaT-mediated regulation of the T3SS in *P. aeruginosa* (**Figure 5**). In addition to its function as a repressor, MvaT was also reported to positively control the expression of the exotoxin A regulatory gene *ptxS* by directly binding to its upstream region in *P. aeruginosa* (Westfall et al., 2004).

Of note, our mass spectrometric analysis also revealed PA3981 as a candidate P*_exsC_* binding protein (**Supplementary Table 3**). PA3981, also named YbeZ, comprises an ATP binding domain and a nucleoside triphosphate hydrolase domain. Our previous study demonstrated that YbeZ controls the T3SS through RetS in *P. aeruginosa* (Xia et al., 2021). Whether YbeZ also regulates T3SS through direct binding to and modulating the P*_exsC_* promoter remains elusive and warrants further study.

Transcription of the *exsA* gene is driven by two distinct promoters, P*_exsA_* and P*_exsC_*. It has been demonstrated that the P*_exsA_* promoter is modulated by several regulatory factors, including activators of Vfr and VqsM and silencers of MvaT and MvaU (Williams McMackin et al., 2019a). Although not binding to and regulating P*_exsA_* promoter activity, ExsA primarily binds to and activates P*_exsC_* promoter activity, leading to a positive feedback loop (Yahr and Frank, 1994). In addition, the P*_exsC_* promoter was also directly bound and stimulated by PsrA, a TetR family transcriptional regulator in *P. aeruginosa* (Shen et al., 2006). Previous studies showed that ExsA binds to the -52 bp region relative to the transcriptional start site of the *exsC* gene (Hovey and Frank, 1995), while the PsrA binding site is located between -207 bp and +44 bp relative to the translational start site (-189 bp to 62 bp relative to the transcription start site) of the *exsC* gene (Shen et al., 2006). In this study, we found that MvaT can also bind to but repress P*_exsC_* promoter activity. According to the EMSA results, the binding site of MvaT on the P*_exsC_* promoter is located between -429 and -380 bp relative to the transcription start site of the *exsC* gene (**Figure 4D**). Since the binding regions of MvaT do not include the -35 or -10 sequences within the P*_exsC_* promoter, the repressive mechanism might not be caused by the occlusion of RNAP by MvaT from the P*_exsC_* promoter. Therefore, the possible repressive mechanism of MvaT on P*_exsC_* is through preventing RNAP from escaping the P*_exsC_* promoter.

Phylogenetic analysis revealed that the T3SS is evolutionarily acquired by horizontal gene transmission (Nguyen et al., 2000). H-NS family DNA-binding proteins play important roles in driving evolution by permitting and regulating horizontally acquired genes (Navarre, 2016). From our experimental results and those of others (Williams McMackin et al., 2019b), both the P*_exsA_* and P*_exsC_* promoters of *exsA* were directly silenced by the MvaT, hinting at the complication of T3SS evolution and regulation.

## Data availability statement

The original contributions presented in the study are included in the article/**Supplementary Material**. Further inquiries can be directed to the corresponding author.

## Author contributions

LY: Investigation, Methodology, Writing – original draft. QL: Investigation, Methodology, Writing – review & editing. XP: Methodology, Formal Analysis, Writing – review & editing. CL: Formal Analysis, Methodology, Writing – review & editing. YB: Formal Analysis, Methodology, Writing – review & editing. FB: Formal Analysis, Funding acquisition, Writing – review & editing. ZC: Formal Analysis, Funding acquisition, Supervision, Writing – review & editing. WW: Formal Analysis, Funding acquisition, Writing – review & editing. UH: Formal Analysis, Writing – review & editing. YJ: Conceptualization, Funding acquisition, Supervision, Writing – review & editing.
