# Supplementary figures and images for: MvaT binds to the P exsC promoter to repress the type III secretion system in Pseudomonas aeruginosa
Source: Front Cell Infect Microbiol. 2023 Nov 6;13:1267748. doi: 10.3389/fcimb.2023.1267748 (PMC10657842; doi:10.3389/fcimb.2023.1267748)

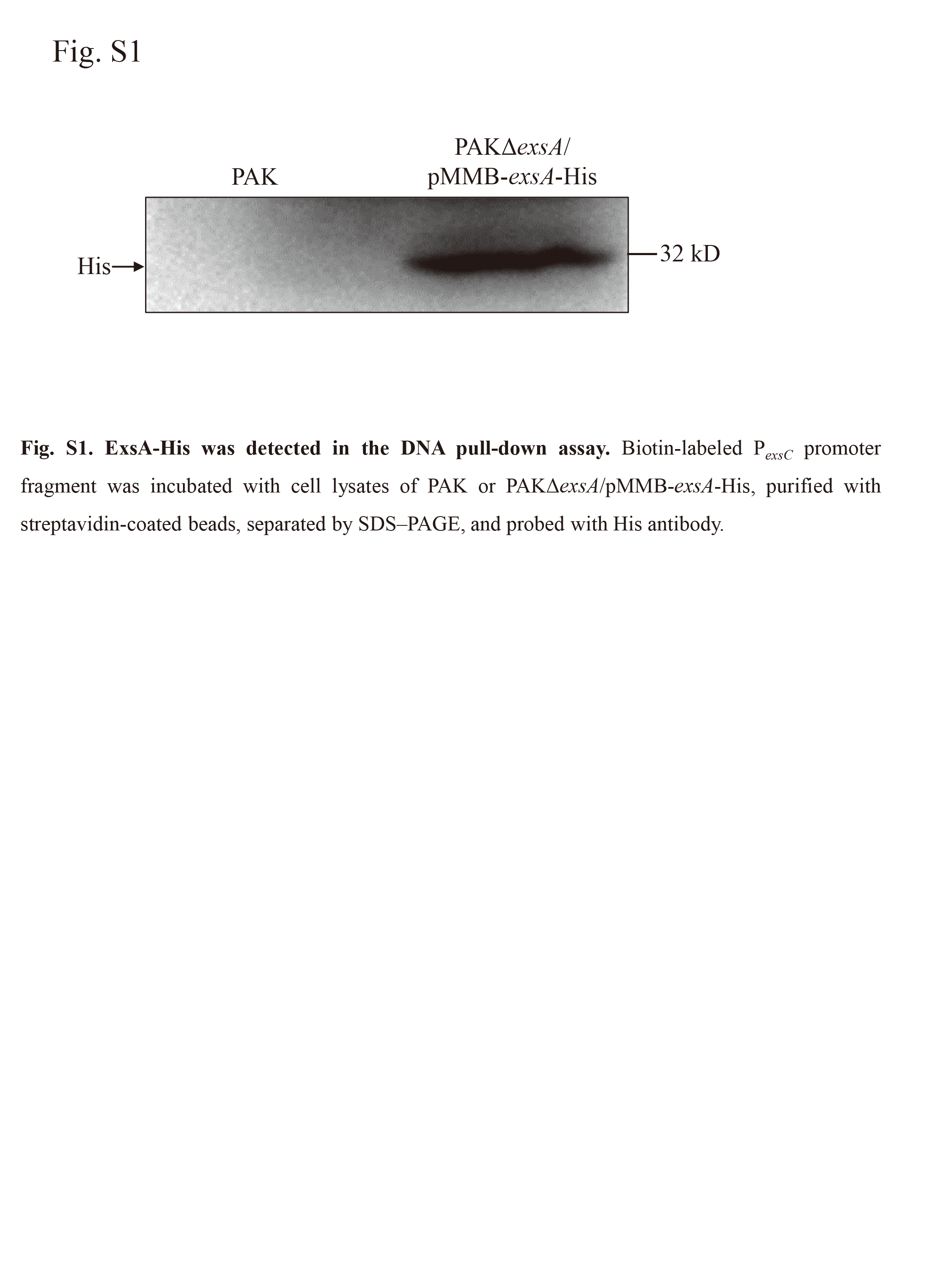

Supplement: Supplementary file 1 [file Image_1.tif]
