# Supplementary material for: MvaT binds to the P exsC promoter to repress the type III secretion system in Pseudomonas aeruginosa
Source: Front Cell Infect Microbiol. 2023 Nov 6;13:1267748. doi: 10.3389/fcimb.2023.1267748 (PMC10657842; doi:10.3389/fcimb.2023.1267748)
Supplement: Supplementary file 2 [file Table_1.docx]

**Table S1.** Bacterial strains and plasmids used in this study.

| Strains or plasmids | Description | Source or reference |
| --- | --- | --- |
| **strains** |  |  |
| DH5α | F^̶^ ϕ 80d*lacZ*∆M15 *endA1 recA1 hsdR17*(r_K_^̶^ m_K_^+^) *supE44 thi-1 relA1* ∆(*lacZYA-argF*)*U169 gyrA96 deoR* | TransGen |
| S17-1 | RP4-2 Tc::Mu Km::Tn*7* Tp^r^ Sm^r^ Pro Res^̶^ Mod^+^ | Stratagene |
| BL21 (DE3) | F^-^ *ompT* *hsdSB (rB-, mB-) gal dcm* (DE3) | invitrogen |
| PAK | Wild-type *P. aeruginosa* strain | David Bradley |
| PAKΔ*exsA* | PAK with *exsA* gene deleted | This study |
| PAKΔ*exsA*Δ*mvaT* | PAK with both *exsA* and *mvaT* genes deleted | This study |
| PAKΔ*mvaT* | PAK with *mvaT* gene deleted | This study |
| **Plasmids** |  |  |
| pUCP20 | Shuttle vector between *E. coli* and *P. aeruginosa*; Amp^r^ | [1] |
| pMMB | Shuttle vector pMMB67EH between *E. coli* and *P. aeruginosa*; Amp^r^ | ATCC |
| pMMB-*exsA*-His | His-tagged *exsA* gene from PAK in pMMB67EH; Amp^r^ | This study |
| pMMB-*mvaT* | *mvaT* gene from PAK in pMMB67EH; Amp^r^ | This study |
| pEX18Tc | Gene knockout vector; Tc^r^ | [2] |
| pEX18Tc-*mvaT* | *mvaT* gene deletion on pEX18Tc; Tc^r^ | This study |
| P*_exsC_*-*lacZ* | *exsC* promoter fused to promoterless *lac*Z on pDN19*lac*ZΩ; Sp^r^, Sm^r^, Tc^r^ | [3] |
| P*_exsC_*_mut_-*lacZ* | *exsC* promoter without MvaT binding region fused to promoterless *lac*Z on pDN19*lac*ZΩ; Sp^r^, Sm^r^, Tc^r^ | This study |
| P*_exoT_*-*lacZ* | *exoT* promoter fused to promoterless *lac*Z on pDN19*lac*ZΩ; Sp^r^, Sm^r^, Tc^r^ | [4] |
| pET28a | expression vector, Kan^r^ | Novagen |
| pET28a-*mvaT* | *mvaT* gene cloned into pET28a expression vector, Kan^r^ | This study |

1. West, S.E., et al., *Construction of improved Escherichia-Pseudomonas shuttle vectors derived from pUC18/19 and sequence of the region required for their replication in Pseudomonas aeruginosa.* Gene, 1994. **148**(1): p. 81-6.

2. Schweizer, H.P., *Allelic exchange in Pseudomonas aeruginosa using novel ColE1-type vectors and a family of cassettes containing a portable oriT and the counter-selectable Bacillus subtilis sacB marker.* Mol Microbiol, 1992. **6**(9): p. 1195-204.

3. Deng, X., et al., *Fis Regulates Type III Secretion System by Influencing the Transcription of exsA in Pseudomonas aeruginosa Strain PA14.* Front Microbiol, 2017. **8**: p. 669.

4. Ha, U. and S. Jin, *Growth phase-dependent invasion of Pseudomonas aeruginosa and its survival within HeLa cells.* Infect Immun, 2001. **69**(7): p. 4398-406.
