# Supplementary material for: MvaT binds to the P exsC promoter to repress the type III secretion system in Pseudomonas aeruginosa
Source: Front Cell Infect Microbiol. 2023 Nov 6;13:1267748. doi: 10.3389/fcimb.2023.1267748 (PMC10657842; doi:10.3389/fcimb.2023.1267748)
Supplement: Supplementary file 3 [file Table_2.doc]

**Table S2**. Primers used in this study.

| Primer^a^ | Sequence 5’-3’ | Use |
| --- | --- | --- |
| *mvaT*-UF | CGAGCTCGGAAACTCGGTTCCGTCCTC | pEX18Tc-*mvaT* |
| *mvaT*-UR | CGGGATCCGTCAGGTACCTTGTCTGTGCTG |
| *mvaT*-DF | CGGGATCCACCAGTCAGTTCCACGAAGAAC |
| *mvaT*-DR | TGCTCTAGATGAAGCTGGAAATGCTCACCG |
| pMMB-*exsA*-F | CGGAATTCATGCAAGGAGCCAAATC | pMMB-*exsA*-His |
| pMMB-*exsA*-R | CCAAGCTTTCAATGGTGATGGTGATGATGGTTATTTTTAGCCCG |
| pMMB-*mvaT*-F | TACTCAGGATCCTTCGCCCTGACTATTATTGAATCTACTT | pMMB-*mvaT*-His |
| pMMB-*mvaT*-R | CCCAAGCTTTTAGTGGTGGTGGTGGTGGTGGCCGAG  CAGGGTGGCCC |
| pET28a-*mvaT*-F | CATGCCATGGTGTCCCTGATCAACGAATA | pET28a-*mvaT*-His |
| pET28a-*mvaT*-R | CCGCTCGAGGCCGAGCAGGGTGGCCC |
| P*_exsC_*-*lacZ*-F | CGGAATTCTCGAGAGCCGCAACGGCGCC | P*_exsC_*_mut_-*lacZ* |
| P*_exsC_*-*lacZ*-R | GGGGTACCGGGGGCGCCTCCTAAAGCTC |
| P*_exsC_*-biot-F | Biotin-GCGCTTGGCAAGACCTCCGACGA | DNA pull-down assay |
| P*_exsC_*-biot-R | TTAAATCCATGGGGGCGCCTCCT |
| P*_exsC_*-F | FAM-GGCGGACCAGGTGCAGGACAGTG | EMSA |
| P*_exsC_*-R | ATACGGCCTGCGAACTCGGCAAGC |
| P*_exsC_*-1-R | TTGACCTCTCCTTCCCGGGCGCT | EMSA |
| P*_exsC_*-2-F | TGCGCCAGGGCGAATCGCAGG | EMSA |
| P*_exsC_*-2-R | ACGCCAGGCCTGGTTATGGCTCT |
| P*_exsC_*-3-F | ATGAAGGACGTCCTGCAGCTCATCC | EMSA |
| P*_exsC_*-3-R | CGCCTCCTAAAGCTCAGCGCATGC |
| P*_exsC_*-4-F | CTCCGCGCGGGAGGAAAAGGC | EMSA |
| P*_exsC_*-5-R | GATCACCGTGTTGGCCACCTGCAG | EMSA |
| P*_exsC_*-6-F | GGCGGACCAGGTGCAGGACAGTGTGGCGTTGCGTGCGGCGGGCCGTGCCT | EMSA |
| P*_exsC_*-6-R | AGGCACGGCCCGCCGCACGCAACGCCACACTGTCCTGCACCTGGTCCGCC |
| P*_exsC_*-7-F | GTGCCTTCGAGAGCCGCAACGGCGCCCTGCAGGTGGCCAACACGGTGATC | EMSA |
| P*_exsC_*-7-R | GATCACCGTGTTGGCCACCTGCAGGGCGCCGTTGCGGCTCTCGAAGGCAC |
| *algD*-F | CAGGGGTGTCGGAGGGACGAACGGTA | EMSA |
| *algD*-R | ACGGCTATTACTTCAGCGCCGAGCAATC |

a: F: forward; R, reverse; U, upstream of specific gene; D, downstream of specific gene; q, qPCR.
